# GESTACOVID Project: Psychological and Perinatal Effects in Spanish Pregnant Women Subjected to Strict Confinement Due to the COVID-19 Pandemic and Their Evolution during De-Escalation

**DOI:** 10.3390/jcm13010248

**Published:** 2023-12-31

**Authors:** Mar Nieto-Tous, Alba Diaz-Martinez, María De-Arriba-García, Alba Roca-Prats, Sara Monfort-Beltrán, María Ivañez-Muñoz, José Alberola-Rubio, Alfredo Perales, Rogelio Monfort-Ortiz

**Affiliations:** 1 Departmento de Obstetricia y Ginecología, Hospital Universitari i Politècnic La Fe, 46026 Valencia, Spainmonfort_isaort@gva.es (R.M.-O.); 2 Centro de Investigación e Innovación en Bioingeniería, Universitat Politècnica de València, 46022 Valencia, Spain; 3 Departmento de Obstetricia y Ginecología, Hospital de Manises, 46940 Manises, Spain; 4 Instituto de Investigación Sanitaria La Fe de Valencia, Hospital Universitari i Politècnic La Fe, 46026 Valencia, Spain; 5 Departamento de Pediatría, Obstetricia y Ginecología, Facultad de Medicina, Universitat de València, 46010 Valencia, Spain

**Keywords:** puerperal, confinement, de-escalation, COVID-19, psychological affection, GHQ-12, distress, Spanish population

## Abstract

The lockdown and de-escalation process following the COVID-19 pandemic led to a period of new normality. This study aimed to assess the confinement impact on the mental health of peripartum women, as their psychological well-being may be particularly vulnerable and thus affect their offspring’s development. A cross-sectional epidemiological study was conducted among women who gave birth during strict confinement (G0) and the new normality period (G1), in which a self-administered paper-based questionnaire assessed 15 contextual factors and the General Health Questionnaire-12 (GHQ-12). For each item, it was verified whether the positive screening rate differed in each confinement phase, and a risk factor study was conducted. For G0, significantly higher positive screening and preterm birth rates were observed in the positive screening group. In the case of G1, maternal age (>35 years), decreased physical activity, and normal weight were found to be protective factors against distress. This study underscores the heightened mental health risk for postpartum women during major psychosocial upheavals (war, economic crisis, natural disasters, or pandemics), along with their resilience as the positive screening rate decreases with the new normality. Findings encourage adopting strategies to identify high-risk women and promote effective measures, such as promoting physical activity.

## 1. Introduction

The COVID-19 pandemic has been a historical moment characterized by the implementation of health policies to promote social distancing to mitigate the virus spread. In Spain, a progressive lift in the restrictions was implemented over the course of the pandemic according to local contexts [1], changing from an initial complete lockdown to a lessening in the restrictive policies. These policies have changed over the course of the pandemic, going from an initial complete lockdown to a lessening in the restrictive policies [2]. In Valencia, Spain, the strict confinement period known as the state of alarm [3] lasted from 14 March to 18 May 2020, and was followed by a de-escalation process that ended by 21 June 2020, in the new normality period, characterized by an almost complete restoration of previous social activity.

Due to the unique characteristics of the restrictive policies, scientific community concern about their possible deleterious effect on mental health has been raised from the outset. In the general population, except for a slight initial deterioration in symptoms of depression and anxiety, evidence shows that most people have been resilient, and mental health has not changed significantly [4]. Interestingly, longitudinal studies [5] suggest that mental health may have been compromised due to a shock effect, especially at the beginning of the pandemic, where it caused negative emotions such as depression or anxiety, leading to a stabilization of the pre-pandemic mental health levels by the end of 2020 or early 2021. Vulnerable groups deserve special attention because of their lower capacity to adapt to stressful situations [6]. This is the case for pregnant and puerperal women, who have a higher prevalence of emotional instability and mental disorders beyond the pandemic [7]. Indeed, some studies [8,9,10] showed an increase in the prevalence of depression and anxiety in this subgroup during the lockdown. Additionally, perinatal women and newborns may require in-depth assessment and additional monitoring, as their stress has been linked to increased preterm birth, lower birth weight, and an increased prevalence of mental disorders in the offspring, among others [11,12].

Little is currently known about puerperal women’s resilience to stressful situations at this particularly vulnerable time in their lives, despite the important impact of poor mental health in the mother on the short- and long-term psychological development of the infant [8]. This subgroup of women in the new normality period is hypothesized to have lower rates of positive screening for mental health disorders, in line with the normal pathway of the stress response, in which initial acute stress with increased symptoms leads to a chronic response with partial improvement of symptomatology [13]. The aim of this study was to assess the mental health resilience of women in the early postpartum period during the alarm state and the recovery phase of the new normality.

## 2. Materials and Methods

### 2.1. Study Design

A cross-sectional epidemiological study was conducted during the strict confinement (G0, 27 April–20 June) and the new normality period (G1, 21 June–20 September) in Valencia. Given that phase 1 of de-escalation was reached in the Valencian community on 18 May, and that the residual effects of containment could last up to a month, 21 June was set as the demarcation date between stages. During this study period, a total of 1634 deliveries were registered at Hospital La Fe. Therefore, to ensure a confidence level of 95% and a precision of 4%, considering that the proportion of positive screenings in our previous work was 58.22% [10] and possible losses of over 50%, the sample size required to be representative of the population was 861.

Participation was voluntary among those of legal age. Puerperal were given a hard copy of the survey [10] the day following their delivery. It was designed ad hoc to collect both contextual information, drawing on key aspects of clinical practice and experiential knowledge, and the psychological state of each respondent. The questionnaire consists of 28 mandatory questions: the first question asks for consent to participate, the next 15 assesses the contextual factors of the participants, and the last 12 corresponds to the GHQ-12, which assesses the psychological impact. The General Health Questionnaire-12 (GHQ-12) has been chosen as a screening tool to identify the confinement-related distress and coping of postpartum women with the pandemic because its effectiveness in identifying and assessing symptoms of psychological disorders has been found to be significant in previous studies [14,15], in addition to the fact that it can serve as an instrument for international comparative studies.

Items on the GHQ-12 were binary scored, where higher scores indicate greater symptom severity. As the cut-off point of this survey is context-dependent [16], the threshold was based on this same population during the pandemic [10]. Thus, questionnaires scoring above 3 were classified as positive screening, while those scoring below were labelled as negative [10].

All participants were asked to sign a written consent form to collect obstetric and neonatal data. The obstetric variables collected were maternal age, maternal obesity, parity, previous abortion, multiple gestations, gestational age, spontaneity or induction of labour, prematurity of delivery, type of delivery termination (vaginal or caesarean), pregnancy and postpartum complications, previous diagnosis of psychiatric pathology, history of gender violence, and maternal smoking. The perinatal variables set consist of foetal weight, Apgar score (1, 5, and 10 min), arterial and venous umbilical cord pH, sex of the newborn, type of breastfeeding (maternal or formula/mixed), and neonatal complications such as admission to the neonatal intensive care unit.

### 2.2. Statistical Analysis

For each of the 28 questions and the 15 obstetric variables collected, a descriptive statistical analysis and a risk factor study were performed after dichotomization. For the former, the chi-square test (*p* < 0.05) was applied to test whether the positive screening rate was significant, differentiating for each stage of confinement (G0 and G1) and screening group. For the latter, the Wald test (*p* < 0.05) was applied using a logistic regression model [17]. Odds ratios have been considered as a measure of effect size [18]. Finally, to assess the internal consistency of the GHQ-12, Cronbach’s alpha has been calculated. All calculations were performed using SPSS 25 statistical software.

## 3. Results

During this study period, a total of 905 surveys were carried out on the first day of the puerperium, corresponding to 55.38% of the puerperal women in the hospital at that period. A total of 406 surveys were conducted before 21 June (G0) and 499 after (G1) among puerperal women, with a loss rate of 3.79% and 5.67%, respectively, due to incompleteness. Considering the GHQ-12, the Cronbach’s alpha is 0.806, so the internal reliability of the questionnaire is considered to be good.

### 3.1. The De-Escalation Effect

After thresholding on the GHQ-12, a higher average total score was found for G0 (t-student, G0: 3.877 ± 2.814, G1: 3.433 ± 2.840, *p* = 0.019), as well as a significantly higher positive screening rate (chi-square, G0: 58.92%, G1: 50.24%, *p* = 0.009). As expected, the average time since the lock-down was significantly higher for G1 (t-student, G0: 65.16 ± 15.76, G1: 141.10 ± 25.78, *p* < 0.001). The responses per question according to the confinement phase and screening outcome are shown in Table 1.

Among the contextual variables, statistically significant differences between G0 and G1 were found in Q10 (confinement rigidity, chi-square, *p* < 0.001), Q11 (physical activity, chi-square, *p* = 0.001), Q13 (mental health status, chi-square, *p* = 0.002), Q14 (when feeling worse started, chi-square, *p* = 0.003) and Q15 (nervousness, chi-square, *p* < 0.001). A significantly higher proportion of smokers (chi-square, *p* = 0.031) and previous diagnosis of psychiatric pathology (chi-square, *p* = 0.005) were found for G0, as well as a lower proportion of induction of labour (chi-square, *p* = 0.001) and maternal breastfeeding (chi-square, *p* = 0.042).

**Figure 1:**
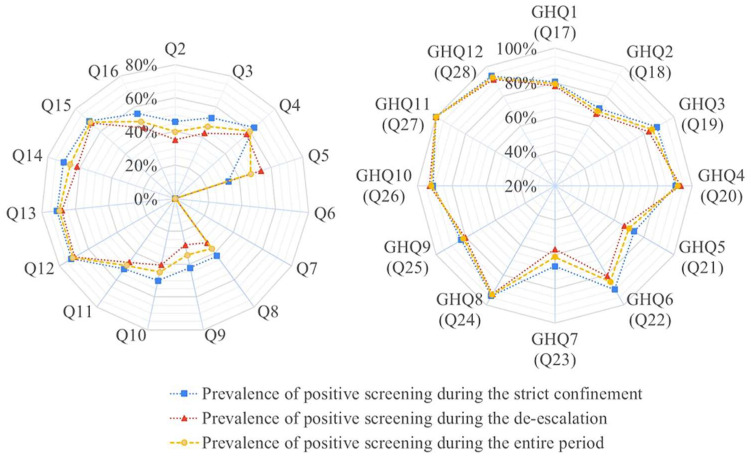
Prevalence of positive screening during the strict confinement, the de-escalation, and the entire period under study for each question of the survey.

In the GHQ-12 analysis, the questions with the highest percentage of positive answers were Q27 (self-esteem, G0: 100%, G1:100%), Q24 (overcoming problems, G0: 94.12%, G1: 92.79%), Q28 (concentration, G0: 93.75%, G1: 91.30%), Q26 (self-confidence, G0: 90.91%, G1: 92.98%) and Q20 (decision making, G0: 90.28%, G1: 93.22%) as can be seen in Figure 1. Significant differences in the proportion of positive responses between G0 and G1 were observed only for questions Q17 (concentration, chi-square, *p* < 0.001), Q20 (decision making, chi-square, *p* = 0.012), and Q21 (overwhelm, chi-square, *p* = 0.014).

### 3.2. Risk Factors

As for the results of the risk factor study, the contextual variables indicate that during the de-confinement process, Q2 (maternal age; G0: 0.787, 95%IC 0.525–1.180; G1: 0.666, 95%IC 0.459–0.966) became a protective factor, whilst Q11 (physical activity as a risk factor; G0: OR 1.660, 95%IC 0.985–2.797; G1: 2.687, 95%IC 1.724–4.188) and Q14 (when feeling worse started; G0: 0.567, 95%IC 0.342–0.939; G1: 0.735, 95%IC 0.453–1.193) became risk factors. Likewise, Q4 (previous diagnosis of COVID; G0: 1.936, 95%IC 1.132–3.314; G1: 2.201, 95%IC 1.362–3.558), Q12 (general health status; G0: 3.407, 95%IC 2.082–5.574; G1: 4.528, 95%IC 2.843–7.210), Q13 (mental health status; G0: 5.056, 95%IC 3.304–7.738; G1: 5.777, 95%IC 3.861–8.645), Q15 (nervousness; G0: 5.922, 95%IC 3.849–9.109; G1: 7.350, 95%IC 4.922–10.975), and Q16 (economical worries; G0: 2.329, 95%IC 1.483–3.659; G1: 2.401, 95%IC 1.544–3.735) were identified as risk factors in both groups. Meanwhile, Q8 (newborn infection overconcerned; G0: 0.430, 95%IC 0.284–0.653; G1: 0.390, 95%IC 0.268–0.566) and Q9 (hospital measures overconcern; G0: 0.598, 95%IC 0.401–0.891; G1: 0.387, 95%IC 0.265–0.564) remained protective factors in both groups. The logistic regression coefficients, along with their 95% confidence intervals and significance levels, for contextual variables distinguishing between G0 and G1, are available in Table S1 (see Supplementary Materials). In terms of clinical variables, preterm birth was no longer a protective factor against distress in G1 as it was in G0.

## 4. Discussion

This cross-sectional study aimed to assess the impact on the mental health and psychological adaptability of puerperal women to the COVID-19 pandemic between strict confinement and the new normality. Spain represents an exceptional enclave for this purpose, given that restrictive policies changed radically after the establishment of the new normality, moving from total isolation to a virtually unrestricted life.

Positive screening prevalence for psychological disorder symptoms is much higher than expected under normal circumstances in both the strict confinement and the new normality groups (G0: 58.92%, G1: 50.24%). Before COVID-19, the generally accepted prevalence for perinatal depression was set at 20% [19], and Van Bussell, evaluating the pregnancy effect by using the GHQ-12, found only a 23.46% positive screening [20]. Furthermore, the finding of a significantly higher positive rate for G0 than for G1 could imply that peripartum women’s mental health symptoms improved over the course of the pandemic and release from restraints, especially since studies in the general population found no evident deterioration of mental health beyond an initial effect [5]. Daly and Robinson described an improvement in symptomatology after an initial increase in psychological distress (March–July 2020) [21]. Similarly, the meta-analysis by Robinson reported that the initial increase in mental health symptoms in the early pandemic (March–April 2020) decreased over time (May–July 2020), when comparing longitudinal studies of mental health symptoms over the course of the pandemic [22]. Moreover, Gimbel reported a decrease in mental health symptomatology over the course of the pandemic in a prospective pregnant and puerperal cohort [13]. In addition, Ceulemans observed a substantial reduction in the prevalence of depressive symptoms (14%, June-July 2020) when compared with studies conducted during earlier stages of the pandemic when strict confinement measures were in place (24–37%, March–May 2020) [23]. Therefore, the symptomatology recovery found in our study seems to be consistent with the stress response pathway, in which a sharp rise in distress is followed by a gradual return to baseline levels [24] and might identify the resilience and adaptability through the pandemic situation in pregnant and puerperal women.

Examining emotion dysregulation in the perinatal period may be useful to identify mothers at risk of psychological distress. Common risk factors for distress in G0 and G1 were previous diagnosis of mental illness (Q4), worsening health status impression (Q12), stress (Q13), increased nervousness (Q15), and financial worries (Q16). Our findings are consistent with previous studies. The history of psychological disorders has been associated with higher anxiety and depression levels during the pandemic in pregnant women in multiple studies [23,25,26]. Psychological well-being has been associated with financial situations [12], especially in middle-income countries where the imposed restrictions made women fear loss of employment or reduced salaries, which affected their sense of security [26,27]. It has been observed that the fears experienced during the COVID-19 pandemic have negative consequences for anxiety levels [28], and nervousness has been described as one of the most common psychological distress symptoms in the nonpregnant population experiencing quarantine during the COVID-19 pandemic [29].

The shared protective factors in G0 and G1 were found to not be overly concerned about the baby’s condition (Q8) and to be worried about the measures taken in the hospital against the spread of the virus (Q9). The Q8 result can be correlated with previous literature in which it has been described that those women who did not fear the vertical transmission of the virus to the baby were protected from anxiety [26]. And a possible interpretation of the Q9 being a protective factor could be that those women who understood the measures taken by the government to avoid the spread of the virus reacted better to the strict hospital measures, which included no visitation and no accompanying, at a vulnerable time as delivery. In addition, it is important to underline that multiple studies have observed a strong correlation between perceived sense of social support and psychological distress in pregnant women [26,30,31].

The added value of our study is the identification of a subgroup of puerperal women at higher risk of maladapting to the pandemic. In our study, maternal age (Q2) became a protective factor, decreased physical activity (Q11) became a risk factor, and when to start feeling worse (Q14) ceased to be significant [10]. Regarding obstetric variables, non-obesity became a risk factor, and preterm birth was no longer significant [10]. To our knowledge, no study has been conducted in this setting aiming to assess the risk factors for this lack of resilience in puerperal women. Interestingly, we found that younger women (<35 years) with normal weight and diminished physical activity were at higher risk. Wu et al. also found that young pregnant women who were, in this case, underweight before pregnancy and who did not do physical exercise were at higher risk of developing depressive and anxiety symptoms during the SARS-CoV-2 outbreak, although they did not examine its adaptability [27]. And Kajdi et al. described that higher maternal age was related to decreased anxiety in middle-income economies [26], maybe due to the age-related maturity. Furthermore, in regard to exercise during pregnancy, high-level evidence, such as one meta-analysis [32] and various systematic reviews [33,34,35], has related it to a significant beneficial effect on depressive and anxious symptoms during pregnancy and puerperium thanks to the release of endorphins, to the point that it is considered an essential factor in the prevention of depressive disorders in women in the postpartum period [36].

The main strength of this study is the extensivity of the survey in the two cohorts of puerperal women, leading to a representative population in both the first confinement and the new normality period. On the other hand, a possible source of bias could be the higher proportion of women with a previous diagnosis of psychiatric pathology in the G0 group, as it has been described as a risk factor for mental health distress in peripartum [37,38]. Another limitation inherent in this type of study was the impossibility of comparing responders with non-responders, which may limit the generalization of results.

## 5. Conclusions

The perinatal period is a critical time of vulnerability to maternal mental health disorders due to external stressors, such as the strict lockdown implemented during the COVID-19 pandemic. This study helps to identify puerperal women at risk of experiencing a mental health deterioration triggered by psychosocial changes through the description of its risk factors: previous mental health illness, worse general health impression, and financial worries. It also demonstrates the resiliency of puerperal women reducing the positive screening prevalence of psychological disorders with the return to the new normality and aids in identifying the women at higher risk of maladaptation: young age (<35 years), normal weight, and lack of physical activity.

The relevant initial impact of the pandemic on puerperal women’s mental health and the absence of the expected recovery in some of them reveal the importance of formulating clinical and public health strategies to detect those pregnant and puerperal women at higher risk of experiencing depression or anxiety during difficult times, such as wars, economic crises, natural disasters, or pandemics. Following our findings, a possible health policy could be the promotion of physical activity in pregnant women, as it may help them cope with external stressors during puerperium.

## Figures and Tables

**Table 1:** Summarized survey responses per question, first entry evaluated as risk factor.

	G0 PS	G0 NS	G0 OR (95%CI)	G1 PS	G1 NS	G1 OR (95%CI)	pS	pG
Q2. Maternal age								
>35 years	68 (7.5%)	80 (8.8%)	0.79 (0.52–1.18)	67 (7.4%)	124 (13.7%)	0.67 * (0.46–0.97)	0.02	0.57
<35 years	134 (14.8%)	124 (13.7%)		138 (15.2%)	170 (18.8%)			
Q3. Living with								
Without children	129 (14.3%)	116 (12.8%)	1.34 (0.90–2.00)	132 (14.6%)	177 (19.6%)	1.20 (0.83–1.73)	0.10	0.63
With children	73 (8.1%)	88 (9.7%)		73 (8.1%)	117 (12.9%)			
Q4. Previous diagnoses								
Diagnosed	43 (4.8%)	25 (2.8%)	1.94 * (1.13–3.31)	47 (5.2%)	35 (3.9%)	2.20 * (1.36–3.56)	<0.01	0.90
No diagnosed	159 (17.6%)	179 (19.8%)		158 (17.5%)	259 (28.6%)			
Q5. Covid-19 diagnoses								
In contact	2 (0.2%)	4 (0.4%)	0.50 (0.09–2.76)	7 (0.8%)	6 (0.7%)	1.70 (0.56–5.13)	0.83	0.24
No	200 (22.1%)	200 (22.1%)		198 (21.9%)	288 (31.8%)			
Q6. Trimester								
Pregnant	6 (0.7%)	6 (0.7%)	-	0 (0.0%)	0 (0.0%)	-	-	-
Puerperal	196 (21.7%)	198 (21.9%)		205 (22.7%)	294 (32.5%)			
Q7. Expected time to delivery								
Pregnant	0 (0.0%)	0 (0.0%)	-	0 (0.0%)	0 (0.0%)	-	-	-
Puerperal	202 (22.3%)	204 (22.5%)		205 (22.7%)	294 (32.5%)			
Q8. Concern newborn affection								
Mostly none	110 (12.2%)	150 (16.6%)	0.43 * (0.28–0.65)	102 (11.3%)	211 (23.3%)	0.39 * (0.27–0.57)	<0.01	0.68
Concerned	92 (10.2%)	54 (6.0%)		103 (11.4%)	83 (9.2%)			
Q9. Concern hospital measures								
Worried	71 (7.8%)	97 (10.7%)	0.60 * (0.40–0.89)	60 (6.6%)	152 (16.8%)	0.39 * (0.26–0.56)	<0.01	0.74
Mostly none	131 (14.5%)	107 (11.8%)		145 (16.0%)	142 (15.7%)			
Q10. Lockdown								
All day	201 (22.2%)	201 (22.2%)	3.00 (0.31–29.08)	191 (21.1%)	281 (31.0%)	0.63 (0.29–1.37)	0.70	<0.01
With exits	1 (0.1%)	3 (0.3%)		14 (1.5%)	13 (1.4%)			
Q11. Physical activity								
Diminished	174 (19.2%)	161 (17.8%)	1.66 (0.98–2.80)	172 (19.0%)	194 (21.4%)	2.69 * (1.72–4.19)	<0.01	<0.01
Not diminished	28 (3.1%)	43 (4.8%)		33 (3.6%)	100 (11.0%)			
Q12. General health								
Worse	71 (7.8%)	28 (3.1%)	3.41 * (2.08–5.57)	73 (8.1%)	32 (3.5%)	4.53 * (2.84–7.21)	<0.01	0.23
Not worse	131 (14.5%)	176 (19.4%)		132 (14.6%)	262 (29.0%)			
Q13. State of mind								
Sadder	128 (14.1%)	52 (5.7%)	5.06 * (3.30–7.74)	117 (12.9%)	55 (6.1%)	5.78 * (3.86–8.64)	<0.01	<0.01
Not sadder	74 (8.2%)	152 (16.8%)		88 (9.7%)	239 (26.4%)			
Q14. Start to feel worse								
Following weeks	81 (9.0%)	61 (6.7%)	0.57 * (0.34–0.94)	87 (9.6%)	74 (8.2%)	0.73 (0.45–1.19)	<0.01	<0.01
First weeks	89 (9.8%)	38 (4.2%)		72 (8.0%)	45 (5.0%)			
Q15. Nervousness								
More nervous	151 (16.7%)	68 (7.5%)	5.92 * (3.85–9.11)	142 (15.7%)	69 (7.6%)	7.35 * (4.92–10.98)	<0.01	<0.01
Equal	51 (5.6%)	136 (15.0%)		63 (7.0%)	225 (24.9%)			
Q16. Economic worries								
Yes	163 (18.0%)	131 (14.5%)	2.33 * (1.48–3.66)	171 (18.9%)	199 (22.0%)	2.40 * (1.54–3.73)	<0.01	0.56
No	39 (4.3%)	73 (8.1%)		34 (3.8%)	95 (10.5%)			

G0: strict confinement group. G1 de-escalation group. PS: positive screening; NS: negative screening. OR (95%CI): odds ratio with 95% confidence interval (*: *p*-value < 0.05). pS: chi-square test’s *p*-value for comparing the screening groups. pG: chi-square test’s p-value for comparing the confinement groups. Comparing the positive and negative screening groups, differences between contextual variables were found in Q2 (extreme maternal age, chi-square, *p* = 0.016), Q4 (previous diagnosis of COVID-19, chi-square, *p* < 0.001), Q8 (newborn infection overconcerned, chi-square, *p* < 0.001), Q9 (hospital measures overconcern, chi-square, *p* < 0.001), Q11 (physical activity, chi-square, *p* < 0.001), Q12 (general health status, chi-square, *p* < 0.001), Q13 (mental health status, chi-square, *p* < 0.001), Q14 (when feeling worse started, chi-square, *p* < 0.001), Q15 (nervousness, chi-square, *p* < 0.001), and Q16 (economical worries, chi-square, *p* < 0.001). No differences were found in the clinical variables.

## Data Availability

Not applicable.

## Supplementary Materials

The following supporting information can be downloaded at: https://www.mdpi.com/article/10.3390/jcm13010248/s1, Table S1. Logistic regression coefficients (β) with 95% confidence intervals (CI) and significance of the coefficients (p-value) for contextual variables discerning between group of strict confinement (G0) and de-escalation (G1). First entry for each question has been evaluated as risk factor.
