# Supplementary material for: GESTACOVID Project: Psychological and Perinatal Effects in Spanish Pregnant Women Subjected to Strict Confinement Due to the COVID-19 Pandemic and Their Evolution during De-Escalation
Source: J Clin Med. 2023 Dec 31;13(1):248. doi: 10.3390/jcm13010248 (PMC10779534; doi:10.3390/jcm13010248)
Supplement: Supplementary file 1 [file jcm-13-00248-s001.zip › jcm-2768271-supplementary.pdf]

Supplementary materials

TableS1. Logistic regression coefficients (β) with 95% confidence intervals (CI) and significance of the coefficients (p-value) for contextual variables discerning between group of strict confinement (G0) and de-escalation (G1). First entry for each question has been evaluated as risk factor.

Label	G0			G1		
	β	*p-value*	*Factor*	*β*	*p-value*	*Factor*
Q2. Maternal age						
> 35 years<br>< 35 years	-0.24<br>(0.52-1.18)	0.25	No signif.	-0.41<br>(0.46-0.97)	0.03	Protector
Q3. Living with						
Without children<br>With children	0.29<br>(0.90-2.00)	0.15	No signif.	0.18<br>(0.83-1.73)	0.34	No signif.
Q4. Previous diagnoses						
Diagnosed<br>No diagnosed	0.66<br>(1.13-3.31)	0.02	Risk	0.79<br>(1.36-3.56)	<0.05	Risk
Q5. Covid-19 diagnoses						
In contact with the virus<br>No	-0.69<br>(0.09-2.76)	0.42	No signif.	0.53<br>(0.56-5.13)	0.34	No signif.
Q6. Trimester						
Pregnant<br>Puerperal	0.01<br>(0.32-3.19)	0.99	No signif.	-	-	-
Q7. Expected time to delivery						
Pregnant<br>Puerperal	-	-	-	-	-	-
Q8. Concern baby affection						
Mostly none<br>Concerned	-0.84<br>(0.28-0.65)	0.00	Protector	-0.94<br>(0.27-0.57)	<0.05	Protector
Q9. Concern hospital measures						
Worried<br>Mostly none	-0.51<br>(0.40-0.89)	0.01	Protector	-0.95<br>(0.26-0.56)	<0.05	Protector
Q10. Lock-down						
All day<br>With exits	1.10<br>(0.31-29.09)	0.32	No signif.	-0.46<br>(0.29-1.37)	0.24	No signif.
Q11. Physical activity						
Diminished<br>Not diminished	0.51<br>(0.98-2.80)	0.06	No signif.	0.99<br>(1.72-4.19)	<0.05	Risk
Q12. General health						
Worse<br>Not worse	1.23<br>(2.08-5.57)	<0.05	Risk	1.51<br>(2.84-7.21)	<0.05	Risk
Q13. State of mind						
Sadder<br>Not sadder	1.62<br>(3.30-7.74)	<0.05	Risk	1.75<br>(3.86-8.64)	<0.05	Risk
Q14. Start to feel worse						
Following weeks<br>First weeks	-0.57<br>(0.34-0.94)	<0.05	Protector	-0.31<br>(0.45-1.19)	0.21	No signif.
Q15. Nervousness						
More nervous<br>Not more nervous	1.78<br>(3.85-9.11)	<0.05	Risk	1.99<br>(4.92-10.98)	<0.05	Risk
Q16. Economic worries						

Yes	0.85 (1.48-3.66)	0.00	Risk	0.88 (1.54-3.73)	0.00	Risk
No						
